# Comparative efficacy of growth factor therapy in healing diabetes‐related foot ulcers: A network meta‐analysis of randomized controlled trials

**DOI:** 10.1002/dmrr.3670

**Published:** 2023-06-05

**Authors:** Shivshankar Thanigaimani, Harry Jin, Usama Ahmad, Raghuveeran Anbalagan, Jonathan Golledge

**Affiliations:** ^1^ Queensland Research Centre for Peripheral Vascular Disease College of Medicine and Dentistry James Cook University Townsville Queensland Australia; ^2^ The Australian Institute of Tropical Health and Medicine James Cook University Townsville Queensland Australia; ^3^ The Department of Vascular and Endovascular Surgery Townsville University Hospital Townsville Queensland Australia

**Keywords:** diabetes‐related foot ulcer, epidermal growth factor, growth factors, platelet‐derived growth factor, protein‐rich plasma

## Abstract

**Introduction:**

This study examined the relative efficacy of growth factor therapies in healing diabetes‐related foot ulcers (DFU).

**Methods:**

PubMed and Cochrane databases were searched for randomized controlled trials testing growth factor therapies for treating DFU. The primary outcome was complete wound closure. Results were reported as relative risk (RR) ± 95% credible intervals (CrI). The risk of bias was assessed using Cochrane's RoB‐2 tool.

**Results:**

A total of 31 RCTs involving 2174 participants were included. Only 13 of the trials (*n* = 924) reported on the aetiology of the ulcers (85.4% neuropathic and 14.6% ischaemic). Epidermal growth factor (RR 3.83; 95% CrI 1.81, 9.10), plasma‐rich protein (PRP) (RR 3.36; 95% CrI 1.66, 8.03) and platelet‐derived growth factor (PDGF) (RR 2.47; 95% CrI 1.23, 5.17) significantly improved the likelihood of complete ulcer healing compared to control. Sub‐analyses suggested that PRP (3 trials ‐ RR 9.69; 95% CrI 1.37, 103.37) and PDGF (6 trials ‐ RR 2.22; 95% CrI 1.12, 5.19) significantly improved the likelihood of wound closure amongst trial mainly recruiting participants with neuropathic ulcers. Eleven trials had a low risk of bias, 9 had some concerns and 11 had a high risk of bias. Sub‐analysis of trials with a low risk of bias suggested that none of the growth factors significantly improved ulcer healing compared with control.

**Discussion:**

This network meta‐analysis found low‐quality evidence that Epidermal growth factor, PRP and PDGF therapy improved DFU healing likelihood compared with control. Larger well‐designed trials are needed.

## 1 INTRODUCTION

Diabetes‐related foot ulcers (DFU) have been estimated to cause about four million years of living with disability.^1^ Treatment of DFU costs approximately US$8659 per patient annually.^2^ Approximately 10 billion US dollars are spent annually on the treatment of DFU.^3^ About 85% of diabetes‐related amputations are preceded by a DFU.^4^ New treatments for DFU are urgently needed.

Growth factors play an important role in promoting wound healing and therefore have been tested as treatments for DFU.^5^ Several meta‐analyses have suggested that the administration of a number of different growth factors significantly improves DFU healing compared with control alone.^6^, ^7^, ^8^, ^9^, ^10^ Despite these findings, The International Working Group of the Diabetic Foot has concluded that the available evidence does not support the use of growth factors in the treatment of DFU.^11^, ^12^ Prior meta‐analyses have mainly focused on the comparison of two treatment strategies such as administration of a growth factor compared to control alone. Network meta‐analysis (NMA) enables the comparison of multiple different treatments. One previous NMA comparing different growth factors suggested that the recombinant human epidermal growth factor (EGF) was the most beneficial growth factor for healing DFU.^13^ However, seven clinical trials have been reported since the publication of this prior NMA.^14^, ^15^, ^16^, ^17^, ^18^, ^19^, ^20^ Therefore, there is a need to perform an updated NMA to assess the evidence for different growth factors in treating DFU.

The aim of this study was to perform an up‐to‐date NMA of randomised clinical trials (RCT) evidence to test the relative efficacy of growth factor therapy in healing DFU in comparison to control.

## 2 METHODS

### 2.1 Search strategy

The systematic review and NMA was performed according to the Preferred Reporting Items for Systematic Review and Meta‐Analysis with an extension for NMA statement^21^ and was registered in the PROSPERO database (Registration Number: CRD42022343029). The literature search and screening were conducted by two authors (ST and HJ). The databases PubMed and Cochrane Central Register for Controlled Trials were searched on 23 May 2022. The full search strategy included terms related to DFU and growth factors (Supplementary Table 1).

### 2.2 Study selection

RCT testing growth factors for treating DFU were eligible for inclusion. Eligible growth factors included recombinant human platelet‐derived growth factor (PDGF), autologous platelet‐rich plasma (PRP), recombinant human EGF, transforming growth factor beta, recombinant human vascular endothelial growth factor (VEGF) and recombinant human fibroblast growth factor (FGF). Control patients were defined as those receiving usual care, best or optimal medical treatment alone, or those receiving placebo. Trials were included only if the growth factor was compared with another type of growth factor or control. Trials which tested multiple different types of growth factors were included only if a control group for each intervention was available. For trials testing multiple doses of one growth factor compared to only one control group, the highest dose growth factor and control groups only were included. Trials were eligible for inclusion if the minimum data (i.e. number of patients with complete wound closure at the end of the study) were published or available from the corresponding author. Trials were included only if all participants had diabetes and a lower limb ulcer. While it was expected that the majority of ulcers would be located in the foot, other lower limb sites were also acceptable. In this study, we defined the cut off to be at a minimum 75% of lower limb ulcers in the foot. When multiple publications arising from the same clinical trial were identified, data from the report with the longest follow‐up were included. Trials published in languages other than English, non‐randomized or crossover trials, observational studies and trials where complete wound closure data were not available were excluded. In addition, studies including participants with infected DFU or people with osteomyelitis were excluded. Eligibility was determined by two authors (ST and HJ), with discrepancies resolved by discussion with the senior author (JG).

### 2.3 Data extraction

Study characteristics, participant risk factors, wound characteristics and primary outcome data were extracted on a customised spreadsheet independently by three authors (ST, HJ and UA). Any inconsistencies were resolved through discussion and confirmed with the senior researcher (JG). The primary outcome was complete wound closure or healing. The following additional data were extracted: Age, sex, body mass index, smoking, Hypertension (HTN), diabetes, Ankle brachial pressure index (ABPI), toe pressure, transcutaneous oxygen pressure, medications, sample size, duration of treatment and duration of follow‐up. In addition, ulcer duration, grade, size and depth were also collected. Additional information from three trials was requested from the corresponding authors^22^, ^23^, ^24^ of whom one author responded with the requested information.^24^

### 2.4 Risk of bias assessment

Two authors (HJ and UA) independently assessed the risk of bias of included trials using the RoB‐2 tool, which assessed key aspects of the reporting including random sequence allocation, allocation concealment, randomisation between intervention groups, deviations from the intended interventions, missing outcome data, the suitability of outcome measurement, blinding of participants and outcome assessors and pre‐specified analysis plan.^25^ The trials were assessed as either at low risk of bias, some concerns (probably low risk of bias), or high risk of bias based on these aspects as per the RoB2 tool. Any inconsistencies were resolved through discussion between the authors until a consensus was reached. No studies were excluded on the basis of risk of bias.

### 2.5 Data analysis

The Bayesian random‐effects NMA was performed using the *R* statistical package “BUGSnet”, which uses the arm based model to assess the geometry of the treatment network and provides effect size estimates for multiple comparisons.^26^ The package develops a random effects model with Bayesian hierarchy using Markov Chain Monte Carlo (MCMC) simulation.^27^ MCMC simulations were run using three chains with different initial values for 100,000 iterations. The model assumes consistency between trials and follows a non‐informative uniform distribution and a weakly informative prior distribution with a variance scaling factor of 2.5. The priors were calculated using the package without any user input using justifications made previously, similar to those used in other NMA packages such as ‘GeMTC’.^28^ The convergence of the resulting model was assessed using the league plots. The fit of the model was assessed by producing leverage plots and a better fit model was selected based on the lowest posterior mean of residual deviance (D_res_). D_res_ is the magnitude of the difference between observed data and that predicted by the model. In a well fit model, D_res_ should be closer to the number of data points (one data point per arm). Inconsistencies within the network model were explored using leverage plots that compare the posterior mean deviance of each data point between the consistency and the inconsistency models. The results of the NMA were reported as relative risk (RR) ± 95% credible intervals (CrI) using the league tables, odds ratio relative to control using forest plots and surface under the cumulative ranking curve (SUCRA) plots. A minimum of 5 trials with a similar method of measurement and reporting was required for any meta‐analysis. Sub‐analyses were performed focused on studies that included participants with largely neuropathic DFUs (85% or above) and trials deemed to be at low risk of bias. A *p*‐value of ≤0.05 was considered statistically significant.

## 3 RESULTS

### 3.1 Study selection

**FIGURE 1:**
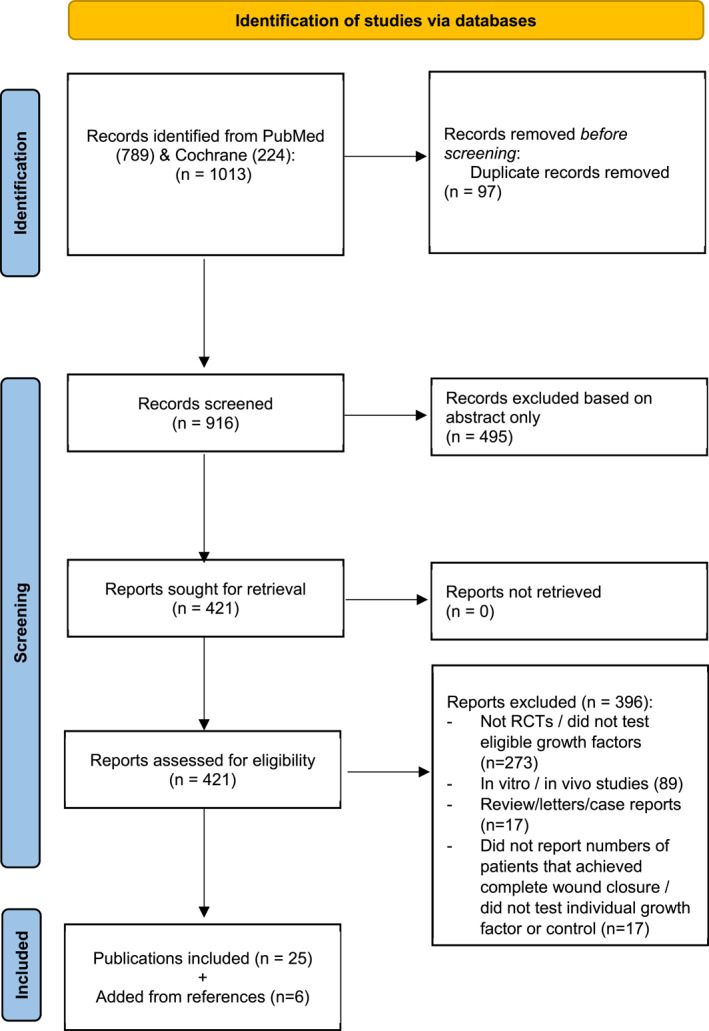
Preferred reporting items for systematic reviews and meta‐analyses flow diagram. A total of 1013 publications were screened and after the exclusion of irrelevant studies, 31 publications were included.

The literature search identified 1013 publications, of which 916 unique records were assessed. Ultimately, 31 RCTs involving 2174 participants were included (Figure 1). All trials compared growth factor therapies against control. Ten trials each tested PDGF^22^, ^23^, ^29^, ^30^, ^31^, ^32^, ^33^, ^34^, ^35^, ^36^ and PRP,^15^, ^18^, ^37^, ^38^, ^39^, ^40^, ^41^, ^42^, ^43^, ^44^ nine trials tested EGF,^16^, ^20^, ^24^, ^45^, ^46^, ^47^, ^48^, ^49^, ^50^ and one trial assessed VEGF^51^ and FGF.^52^ The NMA included control (arms = 31, *n* = 1091), PDGF (arms = 10, *n* = 380), PRP (arms = 10, *n* = 368), EGF (arms = 9, 297), VEGF (arms = 1, *n* = 29) and FGF groups (arms = 1, *n* = 9).

### 3.2 Baseline characteristics of participants

Where reported, participants' age and sex were matched between groups. Characteristics of the included participants were poorly reported (Table 1). None of the included trials reported the prescriptions of diabetes and cardiovascular medications in the recruited participants. A total of 13 trials with 924 participants reported the aetiology of ulcers.^18^, ^23^, ^29^, ^30^, ^33^, ^34^, ^35^, ^36^, ^40^, ^45^, ^46^, ^51^, ^52^ Six trials included a proportion of participants with ischaemic ulcers, which ranged between 7% and 64%.^18^, ^23^, ^29^, ^34^, ^45^, ^46^ The remaining seven trials exclusively recruited participants with neuropathic ulcers.^30^, ^33^, ^35^, ^36^, ^40^, ^51^, ^52^ Overall in these 13 trials, 85.4% had neuropathic ulcers and 14.6% had ischaemic ulcers (Table 2). Twenty‐eight trials reported the ulcer duration^15^, ^16^, ^18^, ^20^, ^22^, ^23^, ^24^, ^30^, ^31^, ^32^, ^33^, ^34^, ^35^, ^36^, ^37^, ^38^, ^39^, ^40^, ^41^, ^42^, ^44^, ^45^, ^46^, ^47^, ^48^, ^50^, ^51^, ^52^ and 29 trials reported ulcer size at the start of the study^15^, ^16^, ^18^, ^20^, ^22^, ^23^, ^24^, ^29^, ^30^, ^31^, ^32^, ^33^, ^34^, ^35^, ^36^, ^37^, ^38^, ^39^, ^40^, ^42^, ^43^, ^44^, ^45^, ^46^, ^47^, ^48^, ^50^, ^51^, ^52^ (Table 2). None of the included trials with mixed aetiology DFU reported complete wound healing of neuropathic and ischaemic ulcers separately. The control provided to control participants in each trial varied as tabulated in Table 2.

**TABLE 1:** Baseline characteristics of participants in the included studies.

Reference	Groups (*n*)	Study period (weeks)	Age (years)	Male gender (%)	Smoking (%)	BMI	ABPI (%)	Toe pressure (mmHg)	Previous amputation (%)	HTN (%)	IHD (%)
Ahmed 2017	Control (28)	12	49.8 ± 15.4	64.3	46	NR	0.85 ± 0.04	NR	NR	89.0	NR
	PRP (28)		43.2 ± 18.2	36.6	61	NR	0.83 ± 0.01	NR	NR	92.0	NR
Afshari 2005	Control (20)	4	59.7 ± 12.3	55.0	45	22.8 ± 3.8	NR	NR	NR	NR	NR
	rhEGF (30)		56.9 ± 12.7	53.3	40	24.0 ± 3.4	NR	NR	NR	NR	NR
Agrawal 2009	Control (14)	12	56.2 ± 8.8	71.4	NR	24.8 ± 3.1	NR	NR	NR	NR	NR
	rhPDGF (14)		54.4 ± 8.8	64.3	NR	26.7 ± 3.0	NR	NR	NR	NR	NR
Bhansali 2009	Control (10)	20	49.5 ± 8.8	50.0	NR	25.29 ± 6.4	1.07 ± 0.10^b^	NR	20.0	NR	NR
							1.10 ± 0.14^c^				
	rhPDGF (10)		51.7 ± 13.6	70.0	NR	22.7 ± 2.8	1.03 ± 0.13^b^	NR	50.0	NR	NR
							1.03 ± 0.13^c^				
Driver 2006	Control (32)	24	57.5 ± 9.1	84.4	NR	NR	NR	NR	NR	NR	NR
	PRP (40)		56.4 ± 10.2	80.0	NR	NR	NR	NR	NR	NR	NR
D’Hemecourt 1998	Control (68)	20	59.6 ± 11.29	79.4	NR	NR	NR	56.5 ± 24.5	NR	NR	NR
	PDGF (34)		58.5 ± 11.9	70.6	NR	NR	NR	49.4 ± 11.9	NR	NR	NR
Elsaid 2020	Control (12)	20	55.6 ± 6.5	50.0	NR	30.7 ± 4.2	NR	NR	NR	33.3	8.3
	PRP (12)		54.7 ± 6.6	66.7	NR	30 ± 5.4	NR	NR	NR	41.7	0.0
Fernandez‐Montequin 2009	Control (48)	8	64.0 (51–70)	56.3	NR	NR	NR	NR	NR	NR	NR
	rhEGF (53)		63 (55–69)	52.8	NR	NR	NR	NR	NR	NR	NR
Gomez‐Villa 2014	Control (16)	8	55.1 ± 10.6	70.6	NR	NR	NR	NR	NR	NR	NR
	rhEGF (16)		62.1 ± 12.8	52.9	NR	NR	NR	NR	NR	NR	NR
Gude 2019	Control (63)	12	66.9^a^	77.8	46.0	NR	NR	NR	NR	NR	NR
	PRP (66)		64.7^a^	77.3	57.6	NR	NR	NR	NR	NR	NR
Gupta 2021	Control (30)	6	55.8 ± 10.2	63.3	NR	NR	NR	NR	NR	NR	NR
	PRP (30)		56.0 ± 9.6	73.3	NR	NR	NR	NR	NR	NR	NR
Hanft 2008	Control (26)	6	59.3 (38–81)	69.2	NR	NR	NR	NR	NR	NR	NR
	rhVEGF (29)		59.5 (42–74)	65.5	NR	NR	NR	NR	NR	NR	NR
Hardikar 2005	Control (58)	10	NR	69.0	NR	NR	NR	NR	NR	NR	NR
	rhPDGF (55)		NR	72.7	NR	NR	NR	NR	NR	NR	NR
Hossam 2022	Control (40)	12	54.8 ± 3.9	85.0	NR	NR	NR	NR	NR	10.0	0.0
	PRP (40)		54.9 ± 2.4	70.0	NR	NR	NR	NR	NR	5.0	0.0
Jaiswal 2010	Control (25)	10	49.9 ± 18.9	92.0	20	NR	NR	NR	4.0	NR	NR
	PDGF (25)		56.2 ± 11.3	76.0	16	NR	NR	NR	4.0	NR	NR
Khandelwal 2013	Control (20)	10	45.0 ± 7.6	55.0	NR	NR	NR	NR	NR	NR	NR
	rhPDGF (20)		43.4 ± 8.1	55.0	NR	NR	NR	NR	NR	NR	NR
Li 2015	Control (55)	12	64.1 ± 9.4	65.5	NR	NR	NR	NR	NR	NR	NR
	PRP (48)		61.4 ± 13.1	62.7	NR	NR	NR	NR	NR	NR	NR
Ma 2015	Control (23)	16	60.1 ± 9.2	100.0	26.0	32.5 ± 7.5	NR	NR	48.0	NR	NR
	rhPDGF (23)		59.3 ± 6.7	100.0	22.0	34.2 ± 8.1	NR	NR	48.0	NR	NR
Malekpour 2021	Control (47)	24	56.7 ± 7.2	63.8	23.4	NR	NR	NR	NR	29.8	NR
	PRP (43)		56.3 ± 7.1	60.4	34.9	NR	NR	NR	NR	34.9	NR
Oliveira 2021	Control (11)	12	65.1 ± 6.5	63.6	NR	32.1 ± 6.3	0.94 ± 0.21	NR	NR	70.0	NR
	rhEGF (14)		60.6 ± 8.6	78.6	NR			NR	NR		NR
Orban 2022	Control (36)	20	59.0 ± 6.7	58.3	NR	NR	NR	NR	NR	NR	NR
	PRP (36)		56.0 ± 8.4	55.6	NR	NR	NR	NR	NR	NR	NR
Park 2018	Control (85)	12	59.3 ± 12.6	57.6	44.7	24.5 ± 4.3	NR	NR	NR	63.5	NR
	rhEGF (82)		56.5 ± 12.7	67.1	54.9	24.4 ± 3.5	NR	NR	NR	56.1	NR
Richard 1995	Control (8)	12	63.6 ± 7.9	87.5	NR	29.3 ± 2.6	NR	NR	NR	NR	NR
	rhbFGF (9)		61.9 ± 10.0	100.0	NR	26.4 ± 4.6	NR	NR	NR	NR	NR
Samuel 2016^d^	Control (14)	24	56.1^a^	58.6	NR	NR	NR	NR	NR	NR	NR
	PDGF (15)				NR	NR	NR	NR	NR	NR	NR
Singla 2014	Control (25)	8	55.8^a^	92.0	NR	NR	NR	NR	24.0	NR	NR
	hEGF (25)		58.8^a^	84.0	NR	NR	NR	NR	32.0	NR	NR
Steed 1995	Control (57)	20	NR	80.7	NR	NR	NR	NR	NR	NR	NR
	rhPDGF (61)		NR	70.5	NR	NR	NR	NR	NR	NR	NR
Tsang 2003	Control (19)	12	64.4 ± 11.7	52.6	NR	25.7 ± 5.2	0.99 ± 0.16	NR	NR	89.5^e^	
	hEGF (21)		62.2 ± 13.7	28.6	NR	23.8 ± 3.2	1.05 ± 0.19	NR	NR	90.5^e^	
Viswanathan 2020	Control (23)	4	55.0 ± 6.8	52.2	NR	NR	NR	NR	NR	NR	NR
	rhEGF (27)		57.9 ± 9.6	55.6	NR	NR	NR	NR	NR	NR	NR
Viswanathan 2006	Control (28)	15	NR	NR	NR	NR	NR	NR	NR	NR	NR
	rhEGF (29)		NR	NR	NR	NR	NR	NR	NR	NR	NR
Wieman 1998	Control (127)	20	58.0 ± 11.8	71.7	NR	NR	NR	55.5 ± 19.6	NR	NR	NR
	rhPDGF (123)		57.0 ± 11.5	66.7	NR	NR	NR	55.0 ± 22.6	NR	NR	NR
Xie 2020	Control (23)	8	61.1 ± 7.9	56.5	NR	NR	NR	NR	NR	NR	NR
	PRP (25)		60.5 ± 8.3	56.0	NR	NR	NR	NR	NR	NR	NR

*Note*: Continuous variables were presented as mean ± SD or median with interquartile range.

Abbreviations: ABPI, Ankle brachial pressure index; BMI, Body mass index; FGF, Fibroblast growth factor; HTN, Hypertension; IHD, Ischaemic heart disease; NR, Not reported; PDGF, Platelet‐derived growth factor; PRP, Plasma‐rich protein; rhEGF, Recombinant human epidermal growth factor; VEGF, Vascular endothelial growth factor.

^a^ SD not reported.

^b^ Right ABPI.

^c^ Left ABPI.

^d^ A total of 29 participants were included in the study, but a groupwise number was not reported. Therefore, the number of ulcers was considered as individual participants for analysis.

^e^ Represents comorbidities including HTN, coronary heart disease and hyperlipidaemia.

**TABLE 2:** Study characteristics.

Reference	Country	Group	Mean ulcer size at recruitment (cm^2^)	Wound duration (weeks)	Neuropathic/Ischaemic ulcer (%)	Control group	Primary outcomes	Complete wound closure (%)
Ahmed 2017	Egypt	Control	2.2 ± 1.2 x 2.6 ± 0.4^e^	11.5 ± 2.8	NR	Daily NS irrigation, povidone iodine 10% ointment, sterile dressing.	Complete wound healing	67.8
		PRP	2.6 ± 0.7 x 2.4 ± 1.1^e^	12.5 ± 1.0				85.7
Afshari 2005^a^	Iran	Control	103.4 ± 147.8	59.7 ± 55.5^c^	93.0/7.0	Daily NS irrigation. Sterile gauze and adhesive tape only	Complete wound healing	10
		EGF	87.5 ± 103.2	42.9 ± 38.4^c^	100.0/0.0			23.3
Agrawal 2009	India	Control	28.7 ± 21.8	NR	85.7/14.3	Daily NS dressing. Debridement as required. Offloading utilised.	Complete wound healing	21.4
		PDGF	54.3 ± 45.2	NR	100.0/0			64.3
Bhansali 2009	India	Control	11.1 ± 9.3	<4= 2; >4 = 8	100.0/0.0	Daily NS dressing. Debridement as required. Offloading utilised.	Complete wound healing	90
		PDGF	18.1 ± 15.9	<4= 2; >4 = 8	100/0.0			90
Driver 2006	England	Control	3.2 ± 3.5	>4	NR	Twice weekly NS gel, contact layer dressing, foam. Debridement as required. Fixed ankle‐foot orthoses utilised.	Complete wound healing	28.1
		PRP	4.0 ± 5.3	>4				32.5
D’Hemercourt 1998	USA	Control	3.5 ± 3.5	>8	100.0/0.0	Dressing changed at 12‐h intervals using moist saline soaked gauze. No weight bearing on the ulcer limb.	Complete wound healing	22.1
		PDGF	2.4 ± 2.0	>8	100.0/0.0			44.1
Elsaid 2020	Egypt	Control	4.0 ± 1.5 x 3.8 ± 1.4^e^	5.58 ± 2.7^d^	66.7/33.3	Daily NS irrigation and dressing changes. Debridement as required.	Percent reduction in the ulcer size	0
		PRP	4.6 ± 2.5 x 5.4 ± 3.4^e^	5.25 ± 3.4^d^	50.0/50.0			25
Fernandez‐Montequin 2009	Cuba	Control	21·8 (8·8–34·6)	4·9 (3·3–12·9)^d^	54.2/45.8	NS gauze dressing. Debridement as required. Offloading utilised. Antibiotics as required.	Granulation tissue covering ≥50% of the ulcer after 2 weeks	52.1
		EGF	28·5 (10·4–42·8)	4·3 (2·9–10·3)^d^	35.4/64.6			75.5
Gomez‐Villa 2014^a^	Mexico	Control	11.9 ± 11.8	36.5 ± 75.8	NR	Ionic silver dressing. Debridement as required. Crutches used for off‐loading.	Complete wound healing	0
		EGF	19.2 ± 15.7	25.8 ± 44.0				23.5
Gude 2019	USA	Control	5.6^a^	NR	NR	Standard of care included the use of semi‐occlusive dressings or hydrocolloid dressings with or without an absorbent dressing.	Complete wound healing	30.2
		PRP	4.1^a^	NR	NR			48.5
Gupta 2021	India	Control	4.96 ± 2.89	11.23 ± 17.69^d^	NR	Weekly NS dressing. Offloading utilised.	Complete wound healing	16.7
		PRP	5.22 ± 3.82	13.7 ± 17.6^d^				20.0
Hanft 2008	USA	Control	1.85^b^	4–24	100.0/0.0	Sterile semi‐permeable barrier and gauze wrap three times per week. Debridement as required. Offloading utilised.	Percent reduction in the ulcer size at day 43	26.0
		VEGF	1.92^b^		100.0/0.0			41.0
Hardikar 2005	India	Control	NR	NR	NR	Daily ulcer cleaning and dressing. Debridement as required. Offloading utilised.	Complete wound healing	31.0
		PDGF	NR	NR	NR			70.9
Hossam 2022	Egypt	Control	14.5 ± 5.6	>6^d^	100.0/0.0	Cleaned and dressed with NS gauze. Debridement as required. Offloading utilised.	Complete wound healing	70 (at week 9)
		PRP	15.2 ± 5.6	>6^d^	100.0/0.0			95 (at week 6)
Jaiswal 2010	India	Control	26.5 ± 2.5	6	32.0/68.0	Local application of KY Jelly daily and wound covered with moist dressing. Pressure off‐loading was carried out in patients with plantar ulcers.	Complete wound healing	60.0
		PDGF	30.0 ± 3.5	5	44.0/56.0			72.0
Khandelwal 2013	India	Control	9.9 ± 5.6	>8	NR	Eusol solution soak for 30 min, hydrogen peroxide and then povidone iodine. Dressed with saline gauze and secondary dressing. Repeated every 24 h. Debridement as required.	Complete wound healing	40.0
		PDGF	19.3 ± 11.3	>8				80.0
Li 2015	China	Control	2.9 (1.0–10.5)	23 (14–60)^c^	NR	Ulcers irrigated and covered with Suile wound dressing	Time to complete wound healing	67.3
		PRP	4.1 (1.4–11.4)	30 (15–90)^c^				85.4
Ma 2015	USA	Control	3.1 ± 3.4	9.8 ± 11.6	NR	Daily NS gauze dressing. Offloading with windowed cast.	Complete wound healing	56.5
		PDGF	2.6 ± 2.7	18.5 ± 22.2				52.2
Malekpour 2021	Iran	Control	NR	>4	NR	Irrigation with NS, dressing it along with silver sulfadiazine ointment twice daily. Debridement as required.	Time to complete wound healing	NR
		PRP	NR	>4				NR
Oliveira 2021	Brazil	Control	10.7 ± 11.1	>12	NR	NS irrigation, sterile gauze, moisturising cream to adjacent skin, crepe bandage. Debridement as required.	Complete wound healing	9.1
		EGF	16.5 ± 15.6	>12				21.4
Orban 2022	Egypt	Control	3.2 ± 1.2	46.7 ± 39.9	NR	NS irrigation, petrolatum gauze and sterile dressing every 2^nd^ day. Debridement as required. Offloading utilised.	Complete wound healing	63.9
		PRP	3.33 ± 1.3	46.6 ± 31.5				86.1
Park 2018	South Korea	Control	2.4 ± 2.7	7.4 ± 15.1^d^	NR	NS spray irrigation, foam dressing twice daily. Debridement as required. Offloading utilised.	Complete wound healing	50.6
		EGF	2.8 ± 3.7	9.6 ± 17.6^d^				73.2
Richard 1995^a^	France	Control	18.1 ± 6.2	27.9 ± 42.2^d^	100.0/0.0	Placebo sprayed onto wound, covered with sterile petrolatum gauze. Debridement as required. Treated daily for the first 6 weeks, twice a week for the following 12 weeks	Wound healing rate	62.5
		FGF	18.0 ± 12.0	22.4 ± 27.9^d^	100.0/0.0			33.3
Samuel 2016	India	Control	31.4 + 61.4	>4	74.2/25.8	Placebo gel with moist NS dressing. Antibiotics and debridement as required.	Complete wound healing	76.5
		PDGF						100.0
Singla 2014	India	Control	21.2^a^	NR	NR	Dressings were performed with betadine daily	Complete wound healing	44.0
		EGF	19.6^a^	NR	NR			88.0
Steed 1995	USA	Control	9.0^b^	>8	100.0/0.0	Placebo gel with moist NS dressing changed every 12 h. Debridement as required.	Complete wound healing	25.0
		PDGF	5.5^b^	>8	100.0/0.0			48.0
Tsang 2003	China	Control	3.5 ± 0.8	12.0 ± 15.5	NR	Treated with Actovegin 5% cream only.	Complete wound healing	42.1
		EGF	3.4 ± 1.1	11.5 ± 14.7	NR			95.3
Viswanathan 2020	India	Control	8.4 ± 7.9	>2	NR	Placebo cream with daily NS dressings performed by patients. Debridement at study commencement.	Complete wound healing	52.2
		EGF	9.1 ± 9.5	>2	NR			77.8
Viswanathan 2006	India	Control	NR	NR	NR	Twice daily gel dressing. Topical and systemic antimicrobials as required.	Complete wound healing	50.0
		rEGF	NR	NR	NR			86.2
Wieman 1998	USA	Control	2.8 ± 4.14	46.0 ± 52.1	100.0/0.0	Placebo gel and twice daily NS dressing performed by patients. Debridement as required. Offloading utilised.	Complete wound healing	34.6
		PDGF	2.6 ± 3.41	46.0 ± 54.7	100.0/0.0			49.6
Xie 2020	China	Control	11.7 ± 7.8	24.3 ± 17.0	NR	Initial debridement was done. Wound was cleaned with saline followed by application of 0.1% rivanor (2‐ethoxy‐6, 9‐aminoacridine lactate) and covered with sterile dressing.	Complete wound healing	87.0
		PRP	11.8 ± 9.7	21.6 ± 18.5	NR			100.0

*Note*: Continuous variables were presented as mean ± SD or median with interquartile range.

Abbreviations: EGF, Epidermal growth factor; FGF, Fibroblast growth factor; NR, Not reported; NS, Normal saline; PDGF, Platelet‐derived growth factor; PRP, Plasma‐rich protein; VEGF, Vascular endothelial growth factor.

^a^ Ulcer size reported in millimetres.

^b^ SD not reported.

^c^ Ulcer duration reported in days.

^d^ Ulcer duration reported in months.

^e^ Ulcer size reported as length x width.

### 3.3 Study characteristics

Ten trials were conducted in India,^15^, ^16^, ^22^, ^23^, ^24^, ^29^, ^30^, ^31^, ^34^, ^49^ six in the United States,^32^, ^33^, ^35^, ^36^, ^43^, ^51^ four in Egypt,^18^, ^37^, ^40^, ^42^ three in China,^39^, ^44^, ^50^ two in Iran^41^, ^45^ and one each in England,^38^ South Korea,^20^ Mexico,^47^ Cuba,^46^ Brazil^48^ and France.^52^ The trial duration ranged from 4 to 24 weeks, with 17 trials having a follow‐up duration between 12 and 20 weeks.^18^, ^20^, ^24^, ^29^, ^30^, ^32^, ^33^, ^35^, ^36^, ^37^, ^39^, ^40^, ^42^, ^43^, ^48^, ^50^, ^52^

### 3.4 Risk of bias of included studies

Overall 11 trials had a low risk of bias,^15^, ^20^, ^23^, ^32^, ^33^, ^36^, ^39^, ^43^, ^47^, ^48^, ^52^ 9 had some concerns,^18^, ^29^, ^35^, ^37^, ^40^, ^41^, ^45^, ^50^, ^51^ and 11 had a high risk of bias^16^, ^22^, ^24^, ^30^, ^31^, ^34^, ^38^, ^42^, ^44^, ^46^, ^49^ (Table 3). In the randomisation process, 22 trials had a low risk of bias^15^, ^16^, ^18^, ^20^, ^23^, ^29^, ^30^, ^32^, ^33^, ^34^, ^36^, ^37^, ^38^, ^39^, ^43^, ^45^, ^46^, ^47^, ^48^, ^50^, ^52^, ^53^ and 9 had some concerns.^22^, ^24^, ^31^, ^35^, ^41^, ^42^, ^44^, ^49^, ^51^ Thirteen trials had a low risk of bias,^15^, ^16^, ^20^, ^22^, ^23^, ^32^, ^33^, ^36^, ^39^, ^43^, ^47^, ^48^, ^52^ 15 had some concerns^18^, ^24^, ^29^, ^30^, ^31^, ^34^, ^35^, ^37^, ^41^, ^42^, ^44^, ^45^, ^50^, ^51^, ^53^ and 3 had a high risk of bias^38^, ^46^, ^49^ in deviation from the intended intervention. Twenty‐two trials had a low risk of bias,^15^, ^18^, ^20^, ^23^, ^30^, ^32^, ^33^, ^34^, ^36^, ^37^, ^39^, ^41^, ^42^, ^43^, ^44^, ^45^, ^47^, ^48^, ^49^, ^50^, ^52^, ^53^ seven had some concerns,^16^, ^24^, ^29^, ^31^, ^35^, ^38^, ^51^ and two had a high risk of bias^22^, ^46^ in missing outcome data. The measurement of outcomes had a low risk of bias in 27 trials,^15^, ^16^, ^18^, ^20^, ^22^, ^23^, ^29^, ^31^, ^32^, ^33^, ^34^, ^36^, ^37^, ^38^, ^39^, ^41^, ^42^, ^43^, ^45^, ^46^, ^47^, ^48^, ^49^, ^50^, ^51^, ^52^, ^53^ and some concerns in 4 trials.^24^, ^30^, ^35^, ^44^ Sixteen trials had a low risk of bias,^15^, ^18^, ^20^, ^23^, ^29^, ^32^, ^33^, ^36^, ^39^, ^43^, ^47^, ^48^, ^50^, ^51^, ^52^, ^53^ 5 had some concerns^35^, ^37^, ^38^, ^41^, ^45^ and 10 had a high risk of bias^16^, ^22^, ^24^, ^30^, ^31^, ^34^, ^42^, ^44^, ^46^, ^49^ in the selection of reported results.

**TABLE 3:** Risk of bias assessed using the revised Cochrane risk‐of‐bias tool for randomized trials (RoB 2).

Reference	Randomisation process	Deviations from the intended interventions	Missing outcome data	Measurement of outcomes	Selection of the reported result	Overall quality assessment
Afshari 2005	Low	Some concerns	Low	Low	Some concerns	Some concerns
Agrawal 2009	Low	Some concerns	Some concerns	Low	Low	Some concerns
Ahmed 2017	Low	Some concerns	Low	Low	Some concerns	Some concerns
Bhansali 2009	Low	Some concerns	Low	Some concerns	High	High
Driver 2006	Low	High	Some concerns	Low	Some concerns	High
D’Hemecourt 1998	Some concerns	Some concerns	Some concerns	Some concerns	Some concerns	Some concerns
Elsaid 2020	Low	Some concerns	Low	Low	Low	Some concerns
Fernandez‐Montequin 2009	Low	High	High	Low	High	High
Gomez‐Villa 2014	Low	Low	Low	Low	Low	Low
Gude 2019	Low	Low	Low	Low	Low	Low
Gupta 2021	Low	Low	Low	Low	Low	Low
Hardikar 2005	Some concerns	Low	High	Low	High	High
Hanft 2008	Some concerns	Some concerns	Some concerns	Low	Low	Some concerns
Hossam 2022	Low	Some concerns	Low	Low	Low	Some concerns
Jaiswal 2010	Low	Some concerns	Low	Low	High	High
Khandelwal 2013	Some concerns	Some concerns	Some concerns	Low	High	High
Li 2015	Low	Low	Low	Low	Low	Low
Ma 2015	Low	Low	Low	Low	Low	Low
Malekpour 2021	Some concerns	Some concerns	Low	Low	Some concerns	Some concerns
Oliveira 2021	Low	Low	Low	Low	Low	Low
Orban 2022	Some concerns	Some concerns	Low	Low	High	High
Park 2018	Low	Low	Low	Low	Low	Low
Richard 1995	Low	Low	Low	Low	Low	Low
Samuel 2016	Low	Low	Low	Low	Low	Low
Singla 2014	Some concerns	High	Low	Low	High	High
Steed 1995	Low	Low	Low	Low	Low	Low
Tsang 2003	Low	Some concerns	Low	Low	Low	Some concerns
Vishwanathan 2020	Low	Low	Some concerns	Low	High	High
Viswanathan 2006	Some concerns	Some concerns	Some concerns	Some concerns	High	High
Weiman 1998	Low	Low	Low	Low	Low	Low
Xie 2020	Some concerns	Some concerns	Low	Some concerns	High	High

### 3.5 Network models

**FIGURE 2:**
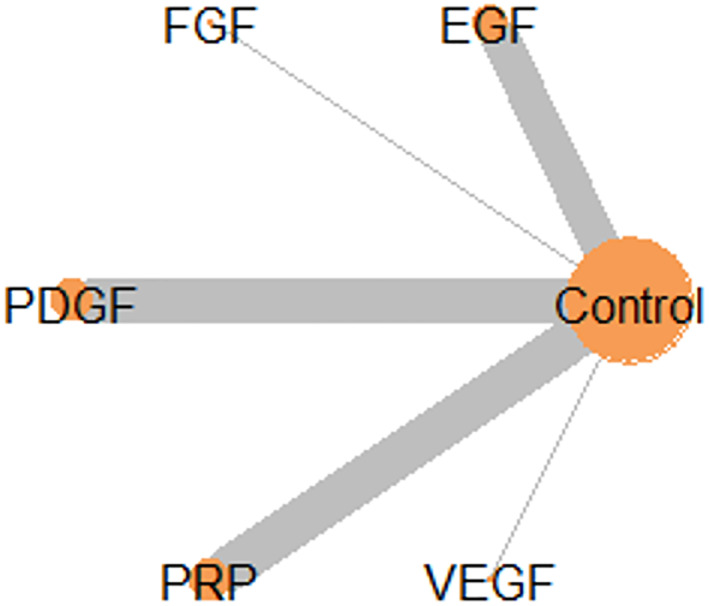
Network plot of all comparisons in the included trials. EGF, Epidermal growth factor; FGF, Fibroblast growth factor; PDGF, Platelet‐derived growth factor; PRP, plasma‐rich protein, and VEGF, vascular endothelial growth factor.

The network plot suggested that the comparison of the primary outcome at different time points was feasible as model convergence was achieved with 100,000 iterations at all follow‐up time points (Figure 2). Leverage plots suggested that a random effects model was a better fit than a fixed effects model (Supplementary Figure 1). Random effects models were therefore applied throughout the analyses. The similarity in posterior deviance of each data point (D_res_) plotted between consistency (63.32) and inconsistency (63.11) models suggested that there were no inconsistencies within the comparisons of the included trials as shown in cross plotting the models (Supplementary Figure 2).

### 3.6 Effect of growth factor therapies on ulcer healing

**FIGURE 3:**
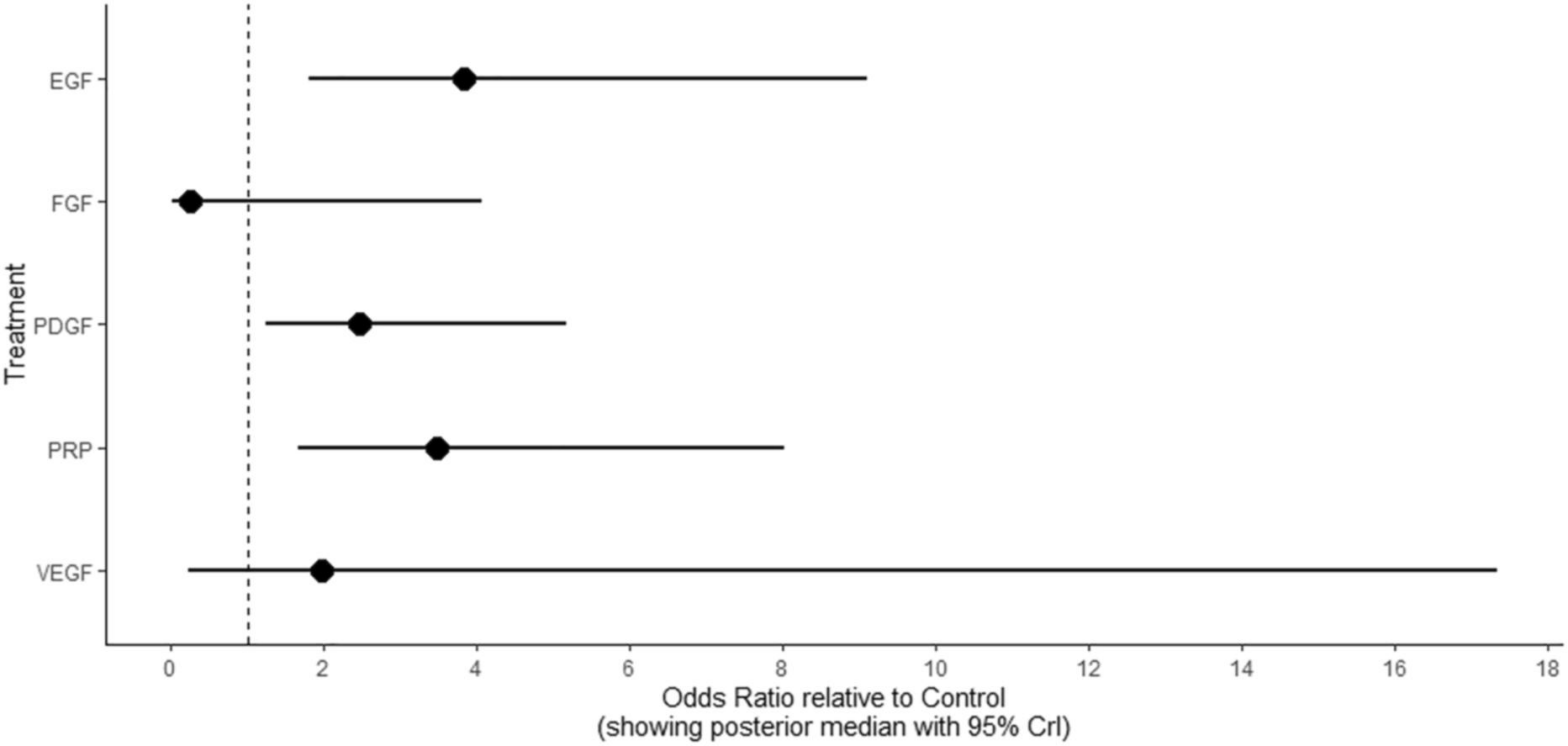
Forest plot showing odds ratio of difference in wound closure of growth factors relative to control. EGF, Epidermal growth factor; FGF, Fibroblast growth factor; PDGF, Platelet‐derived growth factor; PRP, plasma‐rich protein, and VEGF, vascular endothelial growth factor.

**FIGURE 4:**
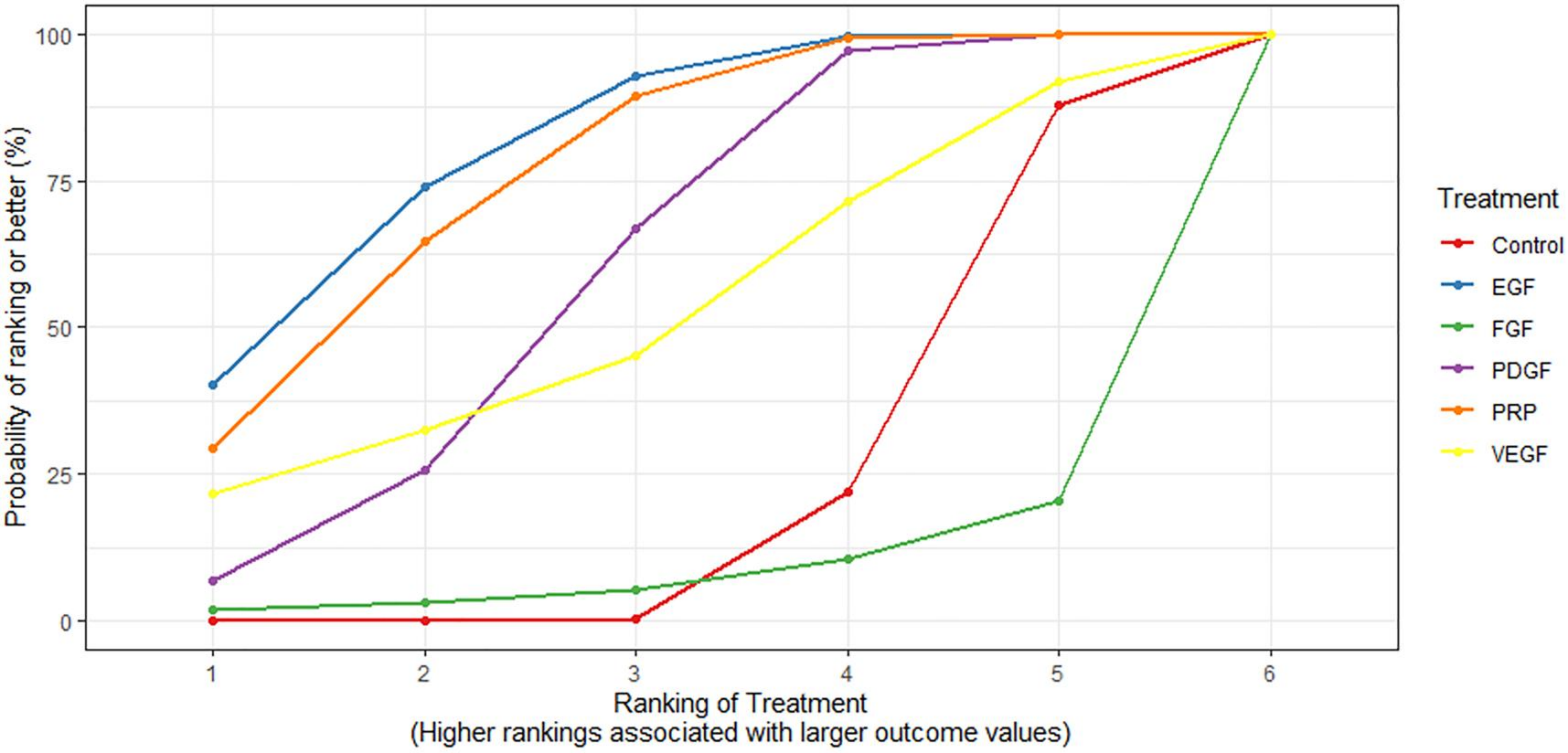
Surface under the cumulative ranking curve (SUCRA) plot showing the probability of the best treatment strategy for attaining wound closure in patients with diabetes‐related foot ulcer within the included trials. EGF, Epidermal growth factor; FGF, Fibroblast growth factor; PDGF, Platelet‐derived growth factor; PRP, plasma‐rich protein, and VEGF, vascular endothelial growth factor.

Compared to control, EGF (RR 3.83; 95% CrI 1.81, 9.10), PRP (RR 3.48; 95% CrI 1.66, 8.03) and PDGF (RR 2.47; 95% CrI: 1.23, 5.17) significantly improved the likelihood of complete ulcer healing (Supplementary Figure 3). Effect estimates with 95% Crl comparing all treatment strategies were provided in the forest plot (Figure 3). Although there were no significant differences in outcomes between the different growth factor therapies, a SUCRA plot suggested that EGF treatment had the best outcome followed by PRP and PDGF (Figure 4).

### 3.7 Network meta‐analysis sub‐analysis testing trials reporting neuropathic diabetes‐related foot ulcers aetiology

A total of 9 trials in which at least 85% of the recruited participants had neuropathic DFUs (370 control and 350 intervention) were included in a sub‐analysis. This suggested that PRP (3 trials ‐ RR 9.69; 95% CrI 1.37, 103.37) and PDGF (6 trials ‐ RR 2.22; 95% CrI 1.12, 5.19) treatment significantly improved the likelihood of complete ulcer healing compared with control (Supplementary Figures 4 and 5). None of the other growth factor therapies significantly improved the likelihood of ulcer healing in comparison to control (Supplementary Figures 4 and 5). Leverage plots suggested that the NMA model in the sub‐analysis was well fit (Supplementary Figure 6).

### 3.8 Network meta‐analysis sub‐analysis of trials with low risk of bias

A total of 11 trials treated as low risk of bias were included in a sub‐analysis. In this subset, 4, 3, 3 and 1 trials assessed the effect of PDGF,^23^, ^32^, ^33^, ^36^ PRP,^15^, ^39^, ^43^ EGF^20^, ^47^, ^48^ and FGF^52^, respectively. The NMA included control (arms = 11, *n* = 489), PDGF (arms = 4, *n* = 222), PRP (arms = 3, *n* = 144), EGF (arms = 3, *n* = 112) and FGF (arms = 1, *n* = 9). A network plot was connected and the NMA model was well fit with no inconsistency (Supplementary Figures 7 and 8). None of the growth factor therapies significantly improved the likelihood of complete ulcer healing in comparison to control (Supplementary Figures 9 and 10).

## 4 DISCUSSION

The results of this NMA of available RCTs suggest that EGF, PRP and PDGF therapy can improve the likelihood of DFU healing in comparison to control. Plasma‐rich protein and PDGF appeared to have the most consistent evidence of efficacy since it was the only growth factor that increased the likelihood of ulcer healing both in the main analysis and the sub‐analysis focused on neuropathic ulcers. Only one study reported neuropathic ulcers in those testing EGF; therefore, its efficacy could not be realistically confirmed. Two thirds of the included trials had some concerns or high risk of bias. Also, where reported, most of the participants had neuropathic ulcers, which typically heal with offloading alone. Since only 105 participants with ischaemic ulcers were identified in the included trials, it was not possible to perform a sub‐analysis focused on ischaemic ulcers. This is a major limitation of the included RCTs as ischaemic ulcers make up the majority of chronic non‐healing DFUs and are much more likely than neuropathic ulcers to be complicated by major amputations, mortality and other complications.^54^ Furthermore, a sub‐analysis restricted to high‐quality trials found that none of the growth factor therapies significantly improved the likelihood of ulcer healing.

Our results are in agreement with a smaller previous NMA, which similarly reported that PRP, EGF and PDGF significantly improved the likelihood of ulcer healing compared to control.^13^ Both NMAs suggest that there was no significant difference in the efficacy of the three growth factor therapies tested. Similar to the previous study which reported that EGF was likely to have the best efficacy, our results were in agreement with EGF having the highest probability of being the best treatment for DFU.^13^ Our results are more updated in comparison to the previous NMA, which included four trials investigating PRP in 113 participants, whereas the current NMA included 10 trials with 368 participants treated with PRP.^13^ Improved wound closure using PRP^10^, ^55^ or EGF^56^ compared to control has been reported in previous conventional meta‐analyses of RCTs as well.

A number of limitations of this NMA and the included RCTs should be noted. The follow up of included participants was relatively short (<24 weeks) and no information on ulcer recurrence was reported. All trials included a small sample size with heterogeneity in terms of ulcer size and duration. This is important as larger and longer term ulcers are less likely to heal^57^ and more likely to be complicated by amputation^58^ and death.^59^ Amputation and mortality data were rarely reported and none of the studies reported information about medications. Furthermore, there was no information reported on modifiable risk factor control, which is an important determinant of outcome.^60^, ^61^ One trial did not report the number of participants who had to be derived from the reported percentage of healed ulcers in relation to the total number of ulcers treated.^23^ One trial crossed over the patients between control and treatment groups after 2 weeks based on responders.^46^ Most importantly, it is rather surprising that none of the trials reported the outcomes for neuropathic and ischaemic DFU independently. Ischaemic ulcers are much less likely to heal and more likely to precipitate major amputation.^54^ For all these reasons, the beneficial effects of growth factors reported in this NMA may not be generalisable to routine clinical practice.^62^ Furthermore, a sub‐analysis of trials deemed to be of low risk of bias suggested that none of the included growth factor therapies were effective in increasing the likelihood of ulcer healing compared to control. Larger well‐designed high‐quality trials are needed for better evaluation of the value of growth factors over control for treating non‐healing ulcers. These should include cost‐effective analyses.

In conclusion, this study suggests that EGF, PRP and PDGF treatments are more effective in healing DFU than control, but these findings could not be replicated in a sub‐analysis restricted to high‐quality trials or those including ischaemic ulcers.

## AUTHOR CONTRIBUTIONS

Shivshankar Thanigaimani was involved in developing the search terms, screening the studies, data extraction, analysis, writing the initial draft and finalising the manuscript. Harry Jin and Usama Ahmad were involved in data extraction and manuscript editing. Jonathan Golledge was involved in conceiving the study, developing the search terms, manuscript writing, critical review of the manuscript and funding acquisition.

## CONFLICT OF INTEREST STATEMENT

None.

### PEER REVIEW

The peer review history for this article is available at https://www.webofscience.com/api/gateway/wos/peer-review/10.1002/dmrr.3670.

## Supporting information

Supplementary Material

## Data Availability

The data that support the findings of this study are available in the supplementary material of this article.
