# Supplementary material for: Comparative efficacy of growth factor therapy in healing diabetes‐related foot ulcers: A network meta‐analysis of randomized controlled trials
Source: Diabetes Metab Res Rev. 2023 Jun 5;39(5):e3670. doi: 10.1002/dmrr.3670 (PMC10909411; doi:10.1002/dmrr.3670)
Supplement: Supplementary file 1 — Supplementary Material [file DMRR-39-e3670-s001.docx]

**Supplementary material**

*Supplementary table 1: Search terms used for in the PubMed and Cochrane databases*

| ***PubMed***  (((foot ulcer) OR (foot disease)) AND (((((((growth factor) OR (platelet)) OR (PDGF)) OR (VEGF)) OR (FGF)) OR (EGF)) OR (PRP))) AND (((wound healing) OR (ulcer healing)) OR (wound closure)) | 789 studies |
| --- | --- |
| ***Cochrane:***  ((growth factor) OR (platelet) OR (PDGF) OR (VEGF) OR (FGF) OR (EGF) OR (PRP)) AND ((Diabetic foot) OR (foot ulcer)) AND ((wound healing) OR (wound closure) OR (Ulcer healing)) in trials. | 224 studies |

**Supplementary figures**


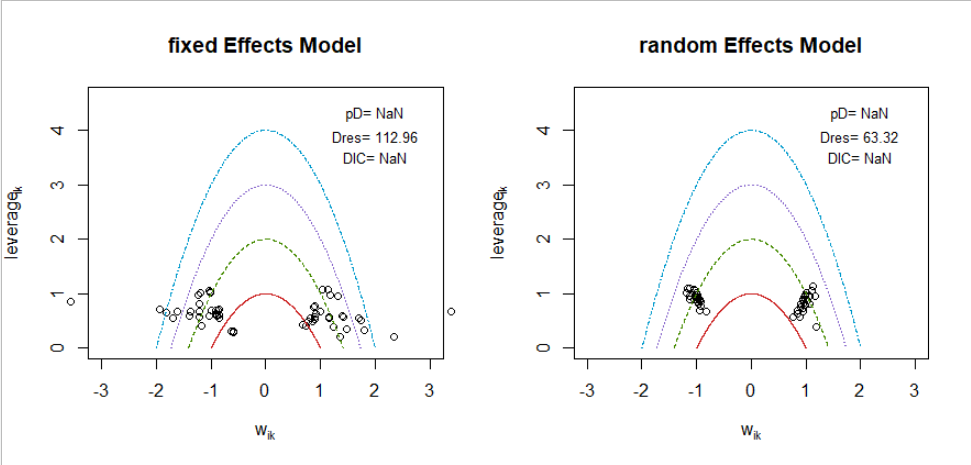


*Supplementary figure 1: Leverage plot demonstrating the assessment of model fitness in fixed and random effects model developed through MCMC simulation of 100,000 iterations. The leverage plot compares the leverage of each data point against their contribution to the total posterior deviance. Points lying outside the purple dotted line in the plots are generally identified as contributing to the model's poor fit. Random effects model showed a better fit with lower D_res_ which indicates posterior mean of residual deviance.*


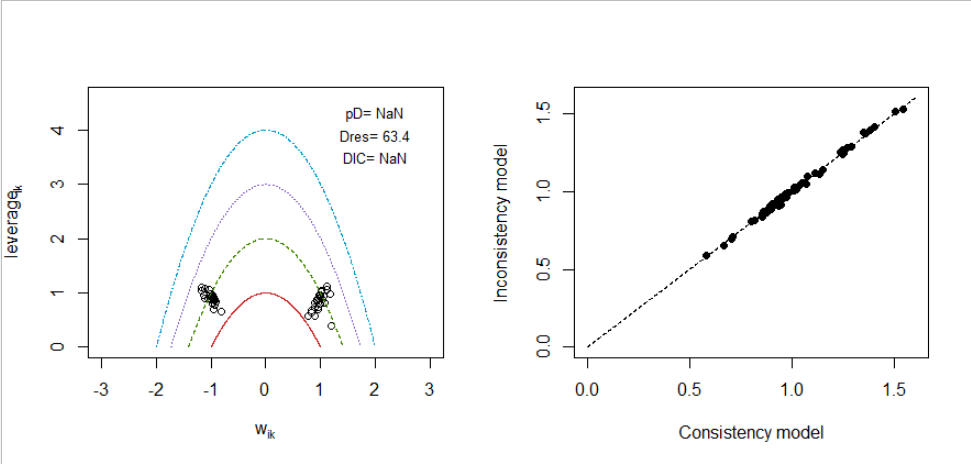


*Supplementary figure 2: Plot of posterior mean deviance of consistency and inconsistency model to identify the loops where inconsistency is present within the included comparisons*


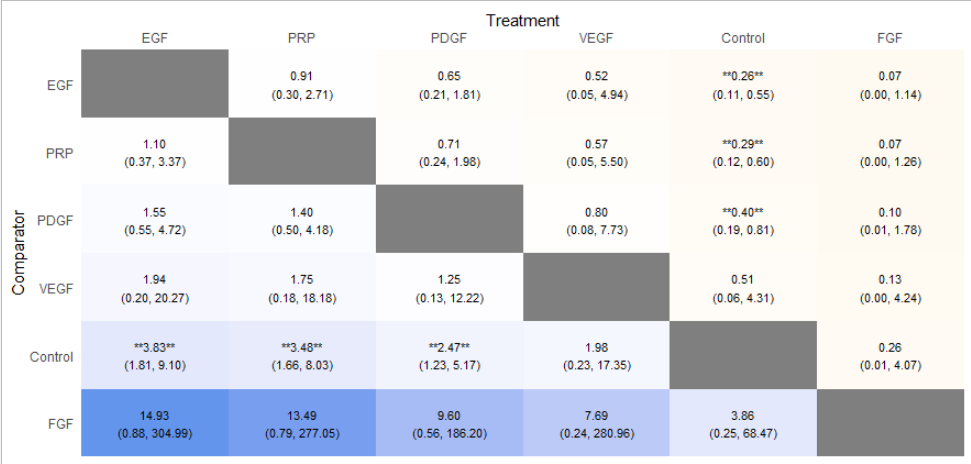


*Supplementary figure 3: League table showing the relative risk ratio with 95% credible intervals in wound closure outcomes between different growth factor therapies in comparison to control group. * indicates statistical significance of p<0.05.*


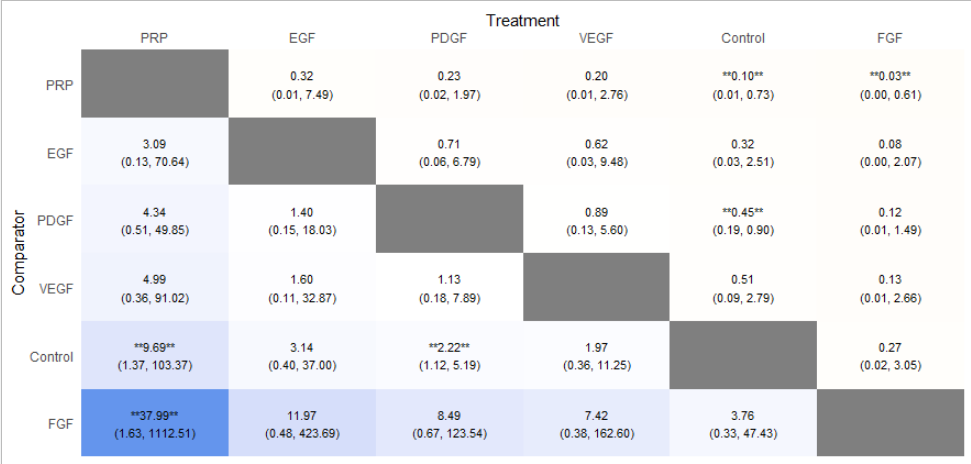


*Supplementary figure 4: League table showing the relative risk with 95% credible intervals in wound closure outcomes between different growth factor therapies in comparison to control in those with neuropathic ulcers only. * indicates statistical significance of p<0.05.*


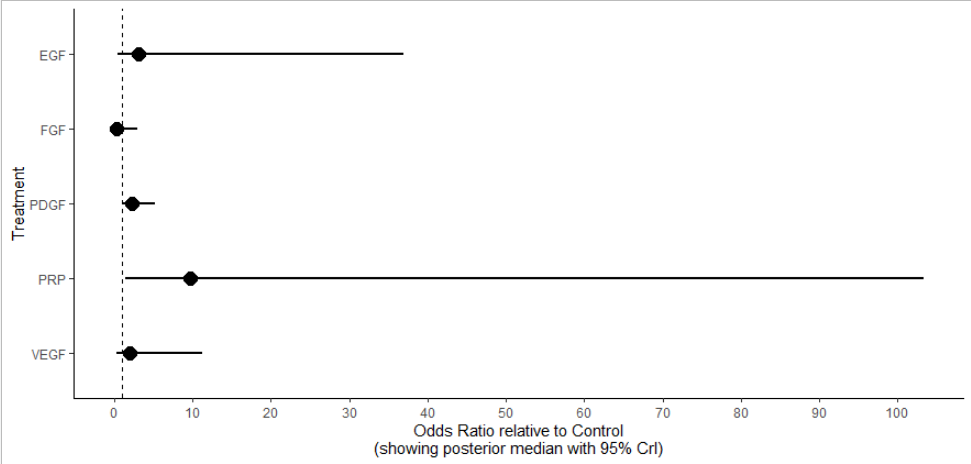


*Supplementary figure 5: Forest plot showing odds ratio of difference in wound closure of growth factor therapies relative to control in those with neuropathic ulcers only.*


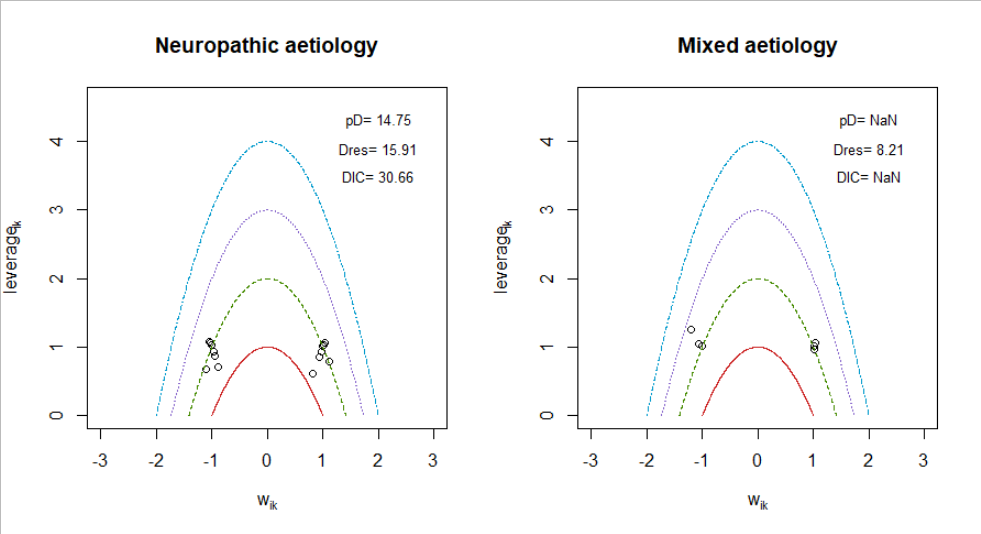


*Supplementary figure 6: Leverage plot showing posterior mean of residual deviance in sub-analysis of trials reporting neuropathic (18 arms) ulcer aetiology.*


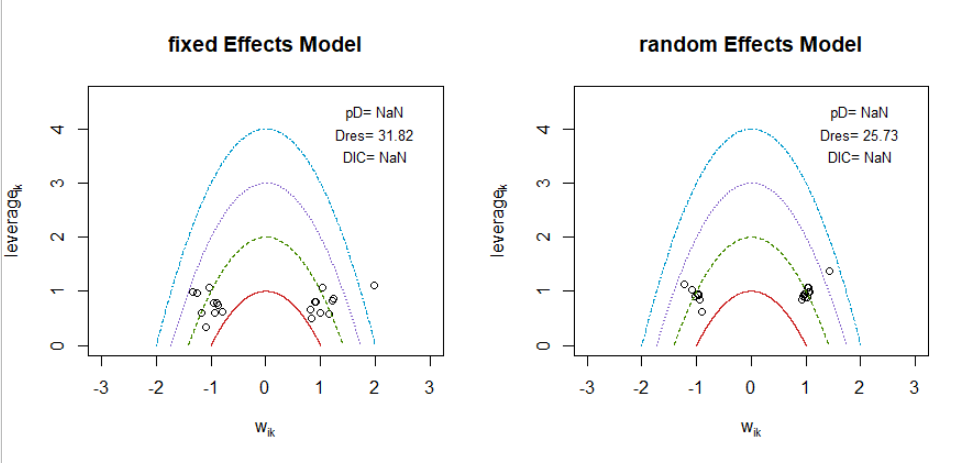


*Supplementary figure 7: Leverage plot showing posterior mean of residual deviance in sub-analysis of trials deemed to be low risk of bias.*


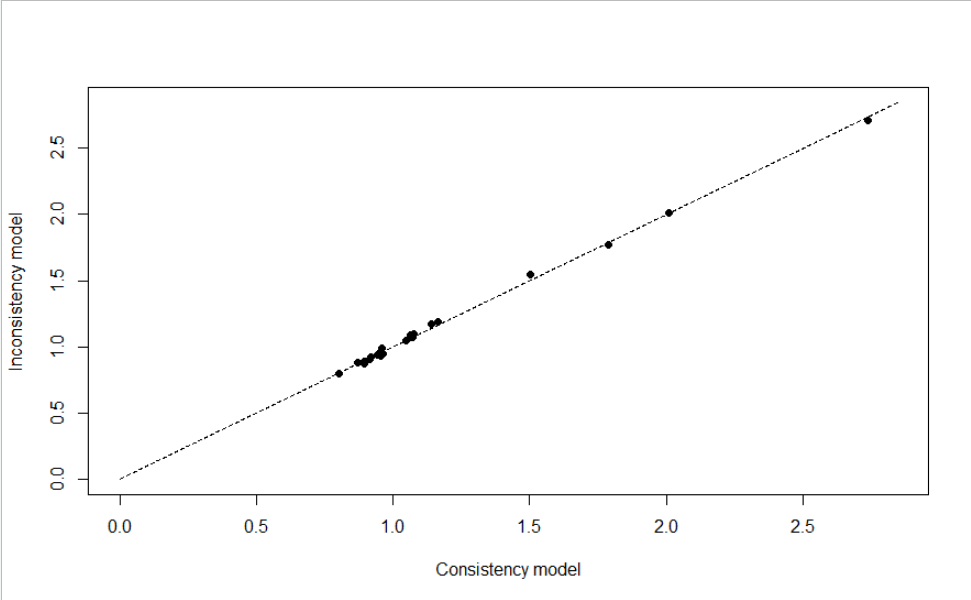


*Supplementary figure 8: Plot of posterior mean deviance of consistency and inconsistency models to identify the loops where inconsistency is present within the included comparisons of trials with low risk of bias.*


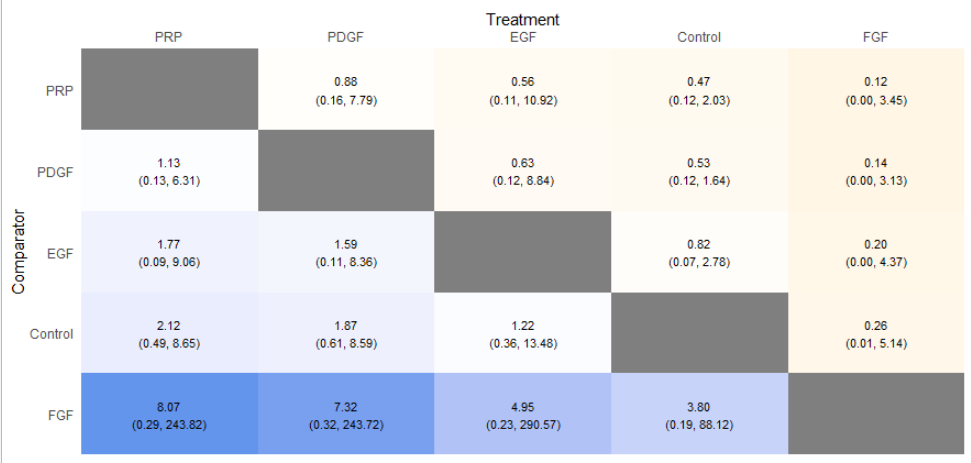


*Supplementary figure 9: League table showing the relative risk with 95% credible intervals in wound closure outcomes between different growth factor therapies in comparison to control in trials deemed to be low risk of bias.*


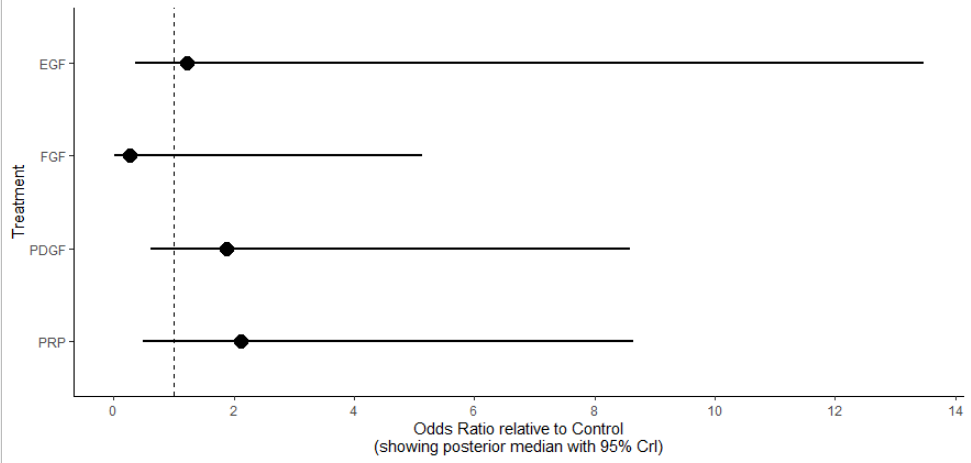


*Supplementary figure 10: Forest plot showing odds ratio of difference in wound closure of growth factor therapies relative to control in trials deemed to be low risk of bias.*
